# An Adaptive Face Tracker with Application in Yawning Detection

**DOI:** 10.3390/s20051494

**Published:** 2020-03-09

**Authors:** Aasim Khurshid, Jacob Scharcanski

**Affiliations:** 1 Sidia Instituto de Ciencia e tecnologia, Amazonas, Manaus 69055-035, Brazil; 2 Instituto de Informatica, UFRGS, Porto Alegre 9500, Brazil; jacobs@inf.ufrgs.br

**Keywords:** face tracking, error prediction, features resyncing, online learning, incremental PCA, yawning detection, feature extraction for emotion analysis

## Abstract

In this work, we propose an adaptive face tracking scheme that compensates for possible face tracking errors during its operation. The proposed scheme is equipped with a tracking divergence estimate, which allows to detect early and minimize the face tracking errors, so the tracked face is not missed indefinitely. When the estimated face tracking error increases, a resyncing mechanism based on Constrained Local Models (CLM) is activated to reduce the tracking errors by re-estimating the tracked facial features’ locations (e.g., facial landmarks). To improve the Constrained Local Model (CLM) feature search mechanism, a Weighted-CLM (W-CLM) is proposed and used in resyncing. The performance of the proposed face tracking method is evaluated in the challenging context of driver monitoring using yawning detection and talking video datasets. Furthermore, an improvement in a yawning detection scheme is proposed. Experiments suggest that our proposed face tracking scheme can obtain a better performance than comparable state-of-the-art face tracking methods and can be successfully applied in yawning detection.

## 1. Introduction

Object visual tracking essentially deals with locating, identifying, and determining the dynamics of moving (possibly deformable) target objects in various areas such as car tracking [1], face detection [2], and driver monitoring [3]. Representational methods are applied successfully for dimensionality reduction and improve discriminative ability in classification problems [4]. Some visual object tracking methods applied representational based methods with pre-computed fixed appearance models [5]; however, the visual appearance of the tracked target object may change along the time and for this reason they may interrupt tracking the target object after a period of time when the tracking conditions change (e.g., the scene illumination changes, occlusions). Some authors proposed to use the data generated during the tracking process to accommodate possible target appearance changes, such as in online learning [6], incremental learning for visual tracking (ivt) [7], patch based approach with online representation of samples [8], and in online feature learning techniques based on dictionaries [1]. Often, online visual tracking methods tend to miss the target object in complex scenarios, such as when the head pose changes while tracking faces, or in cluttered backgrounds and/or in object occlusions [9]. The reasons for this behaviour include the inability to access the tracking error and to update the object appearance at runtime. To approach these issues, Kim et al. [10] utilized a constrained generative approach to generate generic face poses in particle filtering framework, and a pre-trained SVM classifier to discard poorly aligned targets. Furthermore, Correlation filters based methods have become popular in visual object tracking [11]. Li et al. proposed a multi-view model for visual tracking via correlation filters (MCVFT), which fuses multiple features and selects the discriminative features among them [11]. Similarly, Danelljan et al. proposed to use Spatially Regularized Discriminative Correlation Filters (SRDCF) to track visual objects [12]. Furthermore, Discriminant Correlation Filters (DCFs) are also used to train lightweight network architecture to learn the convolutional features for object tracking, such as in DCFNet [13]. Similarly, object tracking by reconstruction based on the online 3D construction of the target to learn DCFs proved to be efficient to track target face with various pose [14]. Moreover, Sanchez et al. [15] proposed an Incremental Cascaded Continuous Regression (iCCR) method for face tracking. The iCCR method is a new formulation for the Cascaded Continuous Regression (CCR) approach, which is adaptive and can be utilized in incremental learning.

On the other hand, geometric shape and appearance models such as Active Appearance Models (AAM) [9], Active Shape Models (ASM) [16] and Constrained Local Models (CLM) [17,18] can capture robust features even in cluttered or fast changing scenarios, improving the robustness of the tracking process. These methods often are based on local shape matching, and try to minimize the difference between a tracked target object and the learned target appearance (i.e., to maximize the shape matching). Unfortunately, most shape and appearance model based methods are not easily applicable to real time tracking due to their complexity. Nevertheless, combining online learning with shape and appearance models can increase the online learning efficiency (e.g., by using appearance models to correct the tracking process and reduce tracking failures).

The proposed approach is applied to face tracking and improves on a well-known object tracking method based on the incremental PCA [7]. The proposed scheme learns from the data generated during face tracking, and corrects the estimated tracking mistakes with a resyncing mechanism. A dynamic tracking error predictor is proposed to estimate how accurately the target face is being tracked. The tracking error predictor adapts itself in time and tends to be consistent in long video sequences (see Section 2.4). If the tracking error is estimated to be increasing, the tracking process is corrected by a resyncing mechanism based on CLM. Furthermore proposed is an improvement of the typical CLM named Weighted CLM (W-CLM) that assigns a weight to each landmark (feature point) based on its consistency in a temporal window (see Section 2.2). In this work, the proposed tracking method is applied to face and facial landmarks tracking, and CLM or W-CLM are used to re-adjust the facial features locations (landmarks) and avoid tracking failures. The proposed Adaptive Face Tracker with Resyncing Mechanism (AFTRM) optimizes the CLM search process without using the landmarks weights. Whereas, another proposed method named Adaptive Face Tracker with Resyncing Mechanism with Weights (AFTRM-W) applies a landmark weight (calculated during the W-CLM training phase) to improve the facial landmark search process. Face tracking based on facial features can provide a low cost solution for a number of measurement applications, such as yawning detection, expression analysis, fatigue detection and vigilance [19]. In this work, the tracked facial landmarks are evaluated in the context of face tracking and in a driving scenario application (i.e, yawning detection).

The major contributions of the paper include:

- Face tracker that can track face and facial landmarks in challenging conditions.
- The proposed tracking scheme utilizes the tracked target face samples collected during tracking to update the appearance model online to adapt to the shape and appearance changes in the tracked face along the time.
- A dynamic error prediction scheme to evaluate the correctness of the tracking process during face tracking.
- Utilization of a resyncing mechanism based on the Constrained Local Models (CLM), when the error predictor indicates high error.
- An improvement in the classical CLM approach, namely Weighted CLM (W-CLM) to improve the facial landmark localization.
- An improvement in a yawning detection scheme by using facial landmarks and imposing multiple conditions to avoid false positives.

The remaining of this paper is organized as follows. The proposed methodology is described in Section 2, followed by our experimental results in Section 3. Finally, Section 4 gives our conclusions and the future prospects of this work.

## 2. Proposed Adaptive Face Tracking Method

**Figure 1:**
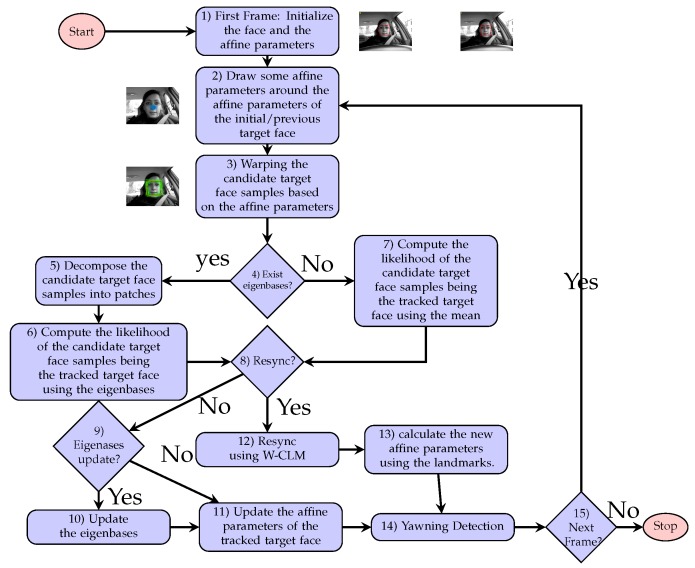
Block diagram of the proposed face tracking approach.

Figure 1 shows the block diagram of the proposed face tracking method, and the blocks functions are explained below:

- Block 1: In the first video frame, the initial target face, its affine parameters and the landmarks are localized using W-CLM (for details on W-CLM, see Section 2.2).
- Block 2: In order to track the target face in the subsequent video frames, new affine parameters values are drawn around the affine parameters values of the initial/tracked target face in the previous video frames (see details in Section 2.3).
- Block 3: The affine parameters previously computed are used to warp the current video frame candidate target face samples of size u×u.
- Block 4: If a specific number (τ) of new target face samples have been gathered, the eigenbases are built.
- Block 5: If the condition in block 4 is satisfied, the candidate target face samples are decomposed into patches (v×v and v≤u), because the eigenbases are built using patches (see Section 2.1).
- Block 6: The tracked target face is found among the candidate target face samples by maximizing the likelihood function in Equation (10) (see details in Section 2.3).
- Block 7: If the condition in block 4 is not satisfied, the tracked target face is estimated by the mean of the previously tracked target face samples. (see Equation (8)).
- Block 8: The proposed error predictor checks if resyncing of the tracked target face is required to correct the tracking process (see details in Section 2.4).
- Blocks 9-10: If resyncing of the tracked target face is not required, the eigenbases are updated if a sufficient new tracked target face samples τ have been accumulated (see details in Section 2.1).
- Blocks 12-13: In case the tracking error is higher than a threshold $\Gamma_{T}$, W-CLM is used to re-locate the tracked target face landmarks and correct the tracking process (see details in Section 2.2).
- Block 14: The yawning is detected (see details in Section 3.4).
- Block 15: The tracked target face and its affine parameters are used as seeds to keep tracking the tracked target face in the next video frame if there are more frames to process.

The proposed tracking algorithm is able to track non-rigid objects such as faces and detect early potential tracking deviations from the tracked target object. The incremental update of the tracking process parameters is inspired on the incremental PCA approach [7]; however, the proposed method uses local texture information (patches of size v×v) rather than global information (the target object as a whole) to build the eigenbases, as explained in Section 2.1. A description of the W-CLM scheme and how it is used as a resyncing mechanism is in Section 2.2. How the proposed tracking method is applied to the face and facial landmark tracking is explained in Section 2.3 and Section 2.4.

### 2.1. Incremental Update of the Eigenbases and the Mean

Let *A* = {I(1),…,I(n)} be a data matrix of d×n dimensions, where each one of the *n* columns I(t) contains a patch of the tracked target face samples represented by a column vector of *d* dimensions, and *n* is the number of samples in *A* (each patch is a target face observation or sample). Let $A\overset{SVD}{=}UCV^{T}$ be the Singular Value Decomposition (SVD) of *A*, where *C* is a diagonal matrix with the singular values (i.e., square root of non-zero eigenvalues), and *U* and *V* are the left and right orthonormal eigenvectors of *A*. Let *B* = {I(1),…,I(m)} of dimensions d×m be the new samples received over time, where *m* is the number of new samples received. Now the goal is to efficiently compute the SVD of the combination of the old data *A* and the new data *B*: $\left[A\;B\right]\overset{SVD}{=}U^{{}^{\prime }}C^{{}^{\prime }}V^{\prime \;T}.$ Computing the SVD every time that new data is received is time-consuming (and impractical) for applications such as object tracking, and the incremental updates of the eigenbases tends to be more interesting. The concatenation of *A* and *B* can be expressed in a partitioned form in a way to utilize the previously computed SVD of *A* as follows [7]:

(1) $$\left[\begin{array}{cc} A & B \end{array}\right]=\left[\begin{array}{c} U\;\tilde{B} \end{array}\right]\left[\begin{array}{cc} C & U^{T}B\\ 0 & {\tilde{B}}^{T}B \end{array}\right]\left[\begin{array}{cc} V^{T} & 0\\ 0 & I \end{array}\right],$$

where $\tilde{B}$ represents the new eigenbases associated to the newly received data matrix *B*, which are orthogonal to the eigenbases in *U* and is unknown at this stage. Let R=$\left[\begin{array}{cc} C & U^{T}B\\ 0 & {\tilde{B}}^{T}B \end{array}\right]$, then $R\overset{SVD}{=}\tilde{U}\tilde{C}{\tilde{V}}^{T}$ can be computed in constant time regardless the initial data size in *A*. Now, the SVD of [AB] can be expressed more conveniently as [7]:

(2) $$\left[\begin{array}{cc} A & B \end{array}\right]\overset{SVD}{=}\left(\begin{array}{c} \left[\begin{array}{cc} U & \tilde{B} \end{array}\right]\tilde{U} \end{array}\right)\tilde{C}\left(\begin{array}{c} {\tilde{V}}^{T}\left[\begin{array}{cc} V^{T} & 0\\ 0 & I \end{array}\right] \end{array}\right),$$

where *I* is the identity matrix. Finally, $U^{{}^{\prime }}$ = [*U* $\tilde{B}$] $\tilde{U}$ and $C^{{}^{\prime }}$ = $\tilde{C}$ are the new eigenvectors (eigenbases) and singular values, respectively, which considers the new data in *B*. Since only $U^{{}^{\prime }}$ and $C^{{}^{\prime }}$ will be utilized in the proposed tracking scheme, $V^{{}^{\prime }}$ is disregarded from now on. Furthermore, only the desired number of eigenvectors (γ) associated with non-zero singular values will be further processed, while other eigenvectors and singular values that exceed the γ ranked singular values will be disregarded. While updating the eigenbases, it is necessary to down-weight the older observations since the more recent observations are more informative about the current appearance of the tracked target face. Therefore, a forgetting factor *f*(∈[0,1]) is multiplied by the singular values in $C^{{}^{\prime }}$ [7], since μ(t) plays a key role in the detection of the tracked target face. Consequently, the mean μ(t) at time *t* is calculated incrementally as follows:

(3) $$\mu \left(t\right)=\frac{f\cdot n\cdot \mu_{n}+m\cdot \mu_{m}}{m+f\cdot n},$$

where $\mu_{n}$ represents the mean of the data matrix *A* with *n* face samples, and $\mu_{m}$ is the mean of the newly added observations *B*, and t=m+n. An important benefit of having the forgetting factor *f* is that the mean μ(t) at time *t* can change in response to new observations, even if the total number of older observations in *A* is large.

### 2.2. Weighted Constrained Local Model (W-CLM) as the Feature Detector Used for Resyncing

The Constrained Local Model method (CLM) tends to be an accurate facial feature detector, but it tends to converge slowly, making its use in tracking problems challenging. Nevertheless, if CLM is used less often in comparison with other components of the tracking process, the CLM based tracking system could be viable for real-time operation. In this work, the proposed tracking scheme is applied to face tracking, and a modified CLM method, namely, the Weighted Constrained Local Model (W-CLM) is utilized to resync important facial features and avoid tracking failure, and also for the initialization of the tracking process. Consequently, the proposed method potentially is self-driven and self-corrected in real-time.

**Weights Computation**: The proposed W-CLM method utilizes CLM training data to evaluate the landmarks consistency by assigning higher weights to more consistent landmarks during the CLM search process. Multivariate Mutual Information (MMI) evaluates the mutual dependence between two or more random variables [20], and is utilized here to evaluate the consistency of each facial landmark. Firstly, MMI is computed independently for the feature vector of each facial landmark within a temporal window. Each feature vector $x_{i}$ represents the texture information in a window of size $\sqrt{l}\times \sqrt{l}$ around a facial landmark location in a given video frame. MMI is used to evaluate the differences of the co-occurrence probabilities of *n* random variables describing the local texture, and indicates how consistent is the texture information around a particular landmark in the training images, and is used as a weight ${\hat{w}}_{i}\in \left[0,1\right]$ of a facial landmark:

(4) $$\begin{array}{c} {\hat{w}}_{i}\left(x_{1},x_{2},,..,x_{N}\right)=log\frac{p(x_{1},x_{2},..,x_{N})}{\prod_{i=1}^{n}p\left(x_{i}\right)}, \end{array}$$

where, $x_{i}$ is a column vector of size *l*, containing texture information around a particular landmark i=1,2,…,Z in a video frame at time *t*. The weights ${\hat{w}}_{i}$ of the landmarks are combined in a diagonal matrix to be used in the W-CLM search process. In practice, the CLM consists of two stages (modules): (1) CLM model building; (2) CLM search [18], that are discussed next:

#### 2.2.1. CLM Model Building

CLM uses two models: (a) a shape model that deals with shape information, and (b) a patch model that considers local patch information. Both models are combined to represent the target object (i.e., face). Images of the cropped faces and a set of facial feature points (landmarks) are used as the training data to build the CLM face model.

In order to build the CLM shape model, all the shapes are aligned with the first (initial) shape of the training set using procrustes analysis [21], which attenuates the adverse effects of shape variations in terms of scale, translation and rotation, leaving only the intrinsic variations of the face shape $S_{r}$. On these aligned faces, the PCA is performed to capture the face shape variations (eigenvectors) in the training data, and to obtain an indication of the total face variation by the eigenvalue of each eigenvector [22]. Therefore, each shape can be written as a linear combination of the eigenvectors *P* and the mean shape ($\bar{S}$) as $S_{r}=\bar{S}+PH_{r}$, where $H_{r}=P^{T}{\hat{S}}_{r}$ is a column vector containing the coefficients of the corresponding eigenvectors *P* for representing the face shape $S_{r}$ in *M*.

In order to build a patch model for each facial landmark, a linear Support Vector Machine (SVM) [23] is trained with positive and negative samples as a patch classifier. The positive examples are the patches (feature templates) captured from the target face only, around the facial landmarks available in the training dataset. The negative examples are the randomly selected patches captured elsewhere in the training images (i.e., excluding the face). Suppose there are *k* training sample vectors $v^{\left(1\right)},v^{\left(2\right)},\ldots v^{\left(k\right)}$, and each training sample vector is a column vector of *l* dimensions $v^{\left(c\right)}={\left[v_{1}^{\left(c\right)},v_{2}^{\left(c\right)},\ldots ,v_{l}^{\left(c\right)}\right]}^{T}$. An input value $u^{\left(c\right)}=\left\{-1,1\right\}$ must be assigned to each training sample c={1,2,…k} of the positive/negative classes which is used as a label of each training sample. The SVM classifier output is written as a linear combination of the input support vectors as $u^{\left(c\right)}=\Omega^{T}v^{\left(c\right)}+\Theta ,$ where $\Omega =\left[\Omega_{1},\Omega_{2},\ldots ,\Omega_{Z}\right]$ are the weights of each dimension of the input support vectors, and Θ is a constant acting as a bias to prevent overfitting. The goal of the SVM training is to search for the right values of weights Ω. For details on training CLM, please refer to [18].

#### 2.2.2. Weighted CLM Search Method

Given a set of initial facial landmarks, a cropped patch around the position of each landmark is classified by the patch model, while preserving the shape constraints, using the following objective function:

(5) $$f\left(S_{t}\right)=\sum_{i=1}^{Z}{\hat{w}}_{i}{ı}_{i}\left(x_{i},y_{i}\right)-\beta \sum_{j=1}^{o}\frac{-h_{j}^{2}}{\lambda_{j}},$$

where, ${ı}_{i}\left(x_{i},y_{i}\right)$ is the patch of size $\sqrt{l}\times \sqrt{l}$ classified by the patch model in the g×g neighborhood of the location of the landmark *i* (g=8 and l=100 in our experiments) and ${\hat{w}}_{i}$ is the weight that describes the impact of the landmark *i* in the optimization process. The term $\sum_{i=1}^{Z}{\hat{w}}_{i}{ı}_{i}\left(x_{i},y_{i}\right)$ in Equation (5) is the patch model response and it is optimized using the quadratic programming and can be readily solved using the Matlab *quadprog* function. The term $\sum_{j=1}^{o}\frac{-h_{j}^{2}}{\lambda_{j}}$ is the shape constraint, where $h_{j}\in H_{r}$ is the corresponding eigenvector coefficient in the eigenvectors representation $H_{r}=P^{T}\hat{S}$ of the current shape *S*, and $\lambda_{j}$ is the eigenvalue corresponding to the eigenvector in *P* and *o* is the number of eigenvectors in *P*, whereas the parameter β∈[0,1] establishes a compromise between the patch and shape models.

For each landmark, the patch model is used to find a response patch at each landmark location in the local region, and the response patch is used to fit a quadratic function. Then, the best landmark positions are obtained by optimizing the function in Equation (5), created by combining the quadratic functions from the patch model and shape constraints from the CLM shape model. Then, each landmark is moved to its new position, and the process is repeated to obtain the optimum landmarks locations (i.e., face shape), or until the maximum number of iterations is reached (see details in [18]). For other promising fitting strategies, please look at the generative shape regularization model for robust face alignment [24] and unified embedding for face recognition and clustering [25]. In case of a new video sequence, the mean face shape $\bar{S}$ is used for the landmarks initialization, but in the subsequent frames the previous frame face landmarks are used to initialize the landmarks.

### 2.3. The Proposed Tracking Method Applied to Human Faces

As mentioned before, in the current work, the proposed tracking method is applied to face tracking. For face tracking, the state at time *t* is described by the affine parameters vector χ(t)= (x(t), y(t), s(t), θ(t), α(t), ϕ(t)), where x(t) and y(t) represent the translation of the tracked target face with respect to the origin of the image, s(t)=M/u is the scale of the tracked target face w.r.t the size of the image (M×N) which contains the tracked target face (u×u), whereas θ(t), α(t) and ϕ(t) are the rotation angle w.r.t the horizontal axis, the aspect ratio, and the skew direction, respectively, at time *t*. The aspect ratio α(t) and the scale s(t) are used to keep the tracked target face in the xy image space (see details in [7]). The dynamics of each parameter in χ(t) are independently modeled by a Gaussian distribution N(.) centered at χ(t−1), and going from χ(t−1) to χ(t) is given by:

(6) p(χ(t)|χ(t−1))=N(χ(t);χ(t−1),ψ(t)),

where ψ(t) is a diagonal matrix with each main diagonal element representing the variance of the corresponding affine parameter. Equation (6) is referred as the motion model, because it models the motion of the tracked target face from one frame to the next frame.

**Figure 2:**
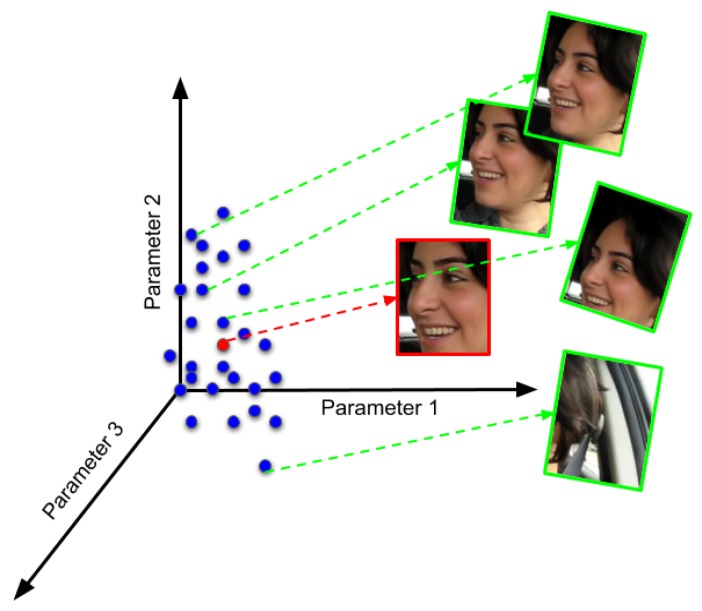
Motion model example (p(χ(t)|χ(t−1))) in image space; Affine parameter space, each point in affine parameter space is warped into a bounding box in image space.

Figure 2 shows an example of the working of the motion model. The affine parameters χ(t) are represented by a point in affine parameter space; the affine parameter space is a six-dimensional space, and only three dimensions are shown in Figure 2. The red point in the Figure 2 represent the affine parameters of the tracked target face in the previous frame. Numerous affine parameters are computed using the Gaussian distribution centered around the affine parameters associated with the tracked target face in the previous frame using Equation (6), and these affine parameters are shown as blue points in Figure 2. Furthermore, these affine parameters are used to warp the candidate target face samples which may contain the tracked target face in the current frame, shown in green color faces in Figure 2 to check if they correspond to the tracked target face I(t).

In order to find the tracked target face in the current frame, every candidate target face sample is represented within the space of the tracked target face I(t) that is spanned by the eigenbases *U* and centered at the mean μ(t), where *U* is obtained incrementally using the method explained in Section 2.1 [7]. The likelihood p(I(t)|χ(t)) that the candidate target face sample is the tracked target face I(t) is inversely proportional to the distance δ of the candidate target face sample to a reference point in the space (i.e., mean μ(t) projected in the space spanned by *U*). This distance is comprised by the sample distance to the space (δt) and the within space distance (δw) of the projected sample to the reference point μ(t). The likelihood ($p_{\delta t}$) that a candidate target face sample projected in the space spanned by *U* corresponds to the tracked target face is approximated by the negative exponential value of δt:

(7) $$\begin{array}{c} p_{\delta t}\left(I\left(t\right)|\chi \left(t\right)\right)=N\left(I\left(t\right);UU^{T}+\varsigma I\right)\approx exp\left(-\delta t\right), \end{array}$$

where, $\delta t=||\left(I\left(t\right)-\mu \left(t\right)\right)-UU^{T}\left(I\left(t\right)-\mu \left(t\right)\right){\left|\right|}^{2}$, ςI is the noise in the observation process, and I is the identity matrix and ideally ς→0. It is worth mentioning that in the initialization, the eigenbases are not available yet, because the eigenbases only are build after a specific number τ of tracked target face samples are observed, then in the initialization *U* = 0 and the mean μ(t) are used to estimate the likelihood p(I(t)|χ(t)) that I(t) contains the tracked target face, and Equation (7) is simplified to:

(8) p(I(t)|χ(t))=exp(−||(I(t)−μ(t))||).

Similarly, the likelihood ($p_{\delta w}$) that I(t) contains the tracked target face is given by the negative exponential of the Mahalanobis distance δw:

(9) $$\begin{array}{c} p_{\delta w}\left(I\left(t\right)|\chi \left(t\right)\right)=N\left(I\left(t\right);\mu \left(t\right),UC^{-2}U^{T}\right)\approx exp\left(-\delta w\right), \end{array}$$

where, $\delta w={||\left(I\left(t\right)-\mu \left(t\right)\right)}^{T}UC^{-2}U^{T}\left(I\left(t\right)-\mu \left(t\right)\right){\left|\right|}^{2}$. Finally, the likelihood of a candidate target face sample I(t) being the tracked target face is given by the combined likelihoods $p_{\delta w}$ and $p_{\delta t}$ to ensure a more reliable decision score as follows:

(10) $$\begin{array}{c} p\left(I\left(t\right)|\chi \left(t\right)\right)=p_{\delta t}\left(I\left(t\right)|\chi \left(t\right)\right)p_{\delta w}\left(I\left(t\right)|\chi \left(t\right)\right). \end{array}$$

The candidate target face sample with the highest likelihood to be the tracked target face in Equation (10) is selected. Furthermore, the affine parameters χ(t) associated with the tracked target face are used to estimate the tracking landmarks locations (facial landmarks) as shown below:

(11) $$\Lambda_{T}\left(t\right)=\chi \left(t\right)\left[\Lambda \left(1\right);\vec{1}\right],$$

where, Λ(1) are the facial landmarks locations in the initial target face and $\vec{1}$ is an unitary vector of length *Z* (total number of landmarks). The pseudo code of the above described procedure is given in Algorithm 1.
**Algorithm 1** Incremental Learning For Face Tracking Algorithm (ILFT).1:**procedure**ILFT(I(t), $\Lambda_{T}\left(t-1\right)$, χ(t−1), *C*, *U*, $\mu_{o}$, I(t−1), flag,Y)                                                          ▹I(t) is the current frame, I(t−1), $\Lambda_{T}\left(t-1\right)$,χ(t−1), are the tracked target face sample, facial landmarks and the affine parameters of the previous frame, *C* and *U* is the singular values and eigenvectors respectively, *C* and *U* are empty matrix in the start and are computed and updated after each τ frames, flag is the counter for number of frames for batch size and Y is 1, if there is at least one more frame to process, otherwise Y is 0.2: 3:    **while** (Y=1) **do**4: 5:        flag←flag+1;6: 7:        Draw a finite number of affine parameters centered at χ(t−1) using Equation (6);8: 9:        Warp the candidate target face samples from I(t) using these affine parameters;10: 11:        Compute the probability of every candidate target face being the tracked target face using Equation (10);12: 13:        Select the candidate target face sample with highest likelihood as the tracked target face sample I(t);14: 15:        Estimate the facial landmarks using Equation (11);16: 17:        **if** (flag≥τ) **then**18: 19:           flag←0;20: 21:           Calculate $C^{{}^{\prime }}$ and $U^{{}^{\prime }}$ (see details in Section 2.1).22: 23:           Update the mean (μ(t)) using Equation (3);24: 25:        **end if**26: 27:        **if** (nextframe == Null) **then**28: 29:           Y←030: 31:        **end if**32: 33:    **end while**34: 35:    **return** $\Lambda_{T}\left(t\right),\chi \left(t\right)$,$C^{{}^{\prime }}$, $U^{{}^{\prime }}$,μ(t),I(t);36: 37:**end procedure**

### 2.4. Tracking Error Prediction and Resyncing Mechanism

Visual tracking is prone to failure if the object changes, moves quickly or changes its appearance. If the tracking methods fails, the tracking error may keep on increasing and the facial tracking process may fail. Most of the methods available do not provide a self assessment of tracking process correctness [5,6,7,26,27]. The proposed method is based on an error predictor that estimates the tracking error ε(t) at runtime. It was found experimentally that a relevant measure to predict the tracking error is the tracking difference of the facial landmarks locations in consecutive frames, which is represented by Δ(t) at time *t*, and its adequacy can be verified by observing the correlation ρ of Δ(t) with the tracking error ε(t), where Δ(t) at time *t* is given by:

(12) $$\begin{array}{c} \Delta \left(t\right)=\frac{1}{Z}\sum_{i=1}^{Z}\left|\right|\Lambda_{T}^{\left(i\right)}\left(t\right)-\Lambda_{T}^{\left(i\right)}\left(t-1\right){\left|\right|}^{2}, \end{array}$$

where, $\Lambda_{T}^{\left(i\right)}\left(t\right)$ is the location $\left(x_{i},y_{i}\right)$ of the facial landmark *i* at time *t* estimated by the proposed method. To further improve the tracking error prediction, a median filter can be applied to the Δ(t) noisy estimates (see details in Section 3).

The next stage of the face tracking process is to predict the potential tracking failures, and if a resyncing is required. This is done by checking if the value of Δ(t) in Equation (12) is higher than a threshold $\Gamma_{T}$. A constant threshold value is not suitable for real applications because Δ(t) may vary from one person to another due to different face sizes, closeness to the camera, and/or the number of facial landmarks used. For this reason, the median value $\left(\Gamma_{T}=Median\left(\Delta \left(T\right)\right)\right)$ is used as a dynamic threshold instead:

(13) $$\Psi \left(t\right)=\left\{\begin{array}{cc} 1, & \mathrm{if}\;\Delta \left(t\right)\geq \Gamma_{T},\\ 0, & \mathrm{otherwise}, \end{array}\right.$$

where Δ(T)={Δ(1),…,Δ(t)}, and Ψ(t) is used to indicate if resyncing is required. Moreover, the proposed error predictor is highly correlated with the actual tracking error (see Section 3). When the tracking predictor indicates a substantial error, i.e., Ψ(t)=1, the W-CLM features are used for correcting (resyncing) the tracking process by re-adjusting the tracked landmarks Λ(t).

Algorithm 2 provides the pseudo code of the proposed method applied to human faces. In the first frame, the face and the facial features are initialized using the W-CLM search method. In the other frames, Algorithm 1 is used to track the face and the facial features until the estimated tracking error increases. When the tracking predictor indicates a substantial error, W-CLM is used to resync the tracking process, which re-locates the facial landmarks $\Lambda_{T}\left(t\right)$ to the correct locations. This error prediction and correction scheme helps the proposed face tracker to adapt to the facial shape and appearance changes of the target along time and the target is not missed indefinitely. Furthermore, the detected facial landmarks $\Lambda_{T}\left(t\right)$ are then used for further processing such as computing the new affine parameters χ(t), and locating the tracked target face based on the candidate samples I(t) in the current frame. Moreover, new eigenbases are created starting from the resynced frame, and the old data is discarded because it is not relevant anymore.
**Algorithm 2** Adaptive Face Tracker with Resyncing Mechanism using W-CLM (AFTRM-W).**procedure**AFTRM(I(t), $\Lambda_{T}\left(t-1\right)$, I(t−1),Y) ▹I(t) is the current frame, I(t−1) and $\Lambda_{T}\left(t-1\right)$ are the tracked target face sample and facial landmarks of the previous frame, respectively and Y indicates if there is atleast one more frame to process, and Y=1 in the first frame.2:     **while** Y=1 **do**4:         Track I(t) and estimate $\Lambda_{T}\left(t\right)$ using Algorithm 1;6:         Compute tracking points difference (Δ(t)) using Equation (12);8:         Calculate Ψ(t) using Equation (13); ▹Ψ(t) results a binary value and checks if resyncing is required.10:         **if** Ψ(t)=1 **then**12:            Update I(t) and $\Lambda_{T}\left(t\right)$ using W-CLM;                                                                     ▹ see Section 2.2.14:            Re-Initialize the eigenbases *U* and the mean μ(t);16:            Calculate the new affine parameters χ(t);18:         **end if**20:     **end while**22:     **return** $\Lambda_{T}\left(t\right)$, χ(t), I(t);24: **end procedure**26: 

## 3. Experimental Results and Discussion

The YawDD [28] and Talking Face Video [29] datasets are used in the experimental evaluation. YawDD dataset contains videos of drivers performing various tasks such as talking/singing and yawning. The camera was installed on the dash or under the car front mirror. The videos were taken under various illumination conditions. YawDD dataset contains the total of 119 participants from different age groups with the minimum age of sixteen years are involved. The videos from 29 participants are recorded using camera installed on the dash, and for other 90 participants, the camera is installed under the front mirror. On the other hand, the Talking Face video consist of 5000 frames obtained from a video of a person engaged in conversation with various face movements [29].

Table 1 shows the important parameters used in the proposed tracking method, their range and optimal values are presented, which are chosen empirically. For building the eigenbases *U*, the candidate/tracked target face sample is resized to u×u (u=32) for computational efficiency, the number of eigenvectors γ = 16, the patch size is set to v×v (v=8) and the eigenbases are updated every five frames (τ=5), with a forgetting factor *f* = 0.95.

The proposed face tracking algorithm is quantitatively evaluated using Center Location Error (CLE), that measures the distance between center locations of the tracked target face with the manually labeled center location of the target face that is used as the groundtruth. Furthermore, for detailed evaluation on YawDD dataset, six videos have been annotated manually, which includes the target face and landmarks (Z=68) on the face, nose and the eyes. These videos contain different background and varied illumination. Additionally, person-specific characteristics, such as face changes, head motion, and glasses are also included. The proposed face tracking method is tested to verify if they can track the facial landmarks consistently on these videos. Hence, the error was measured by the root mean squared error (RMSE) between the estimated landmark locations ($\Lambda_{T}$) and the manually-labeled groundtruth ($\Lambda_{G}$) locations of the landmarks as follows:

(14) $$\varepsilon \left(t\right)=\frac{1}{Z}\sum_{i=1}^{Z}\left|\right|\Lambda_{G}^{\left(i\right)}\left(t\right)-\Lambda_{T}^{\left(i\right)}\left(t\right){\left|\right|}_{2},$$

where ε(t) represents the tracking error in the video frame at time *t*, whereas $\Lambda_{G}^{\left(i\right)}$ represents the ground truth location $\left(x_{i},y_{i}\right)$ of the landmark *i*.

### 3.1. Choice of Batch Size

**Figure 3:**
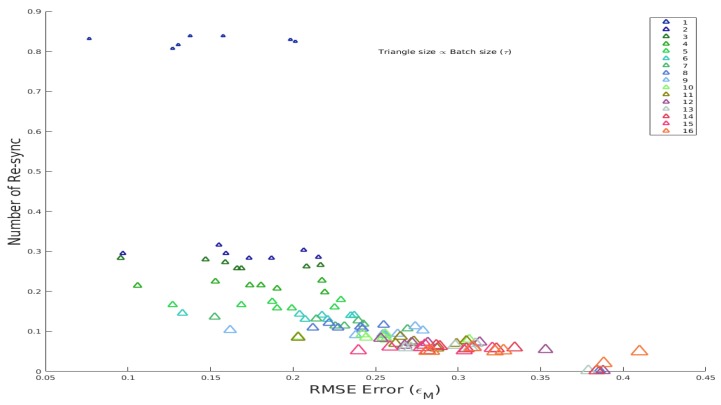
Batch size effect on error (ε) and number of resyncs (r) in multiple videos (normalized to [0,1]).

In the object tracking methods that learn the appearance of the tracked target object incrementally, the batch size plays an important role. Batch size describes that after how many frames the appearance model is updated. Different batch sizes have been tested to optimize the performance of the proposed tracking method. The phenomenon of batch size τ with the average RMSE tracking error $\varepsilon_{M}$ and number of resyncs *r* is shown in Figure 3 for different batch sizes (1≤τ≤16). The size of the triangle indicates the batch size, which means after how many frames the resync of the features is performed (larger the size of the triangle, bigger batch size). A larger batch size (big triangles in Figure 3) requires a lower number of resyncs, but it confers higher errors and vice versa. Contrarily, small triangles tend to lie on the upper left (upper for a large number of resyncs and left confers to smaller error) of the plot, which shows that more resyncs are required, and the error is low. However, frequent resyncs and updates may slow down the number of frames processed per second, as shown in Table 2.

**Figure 4:**
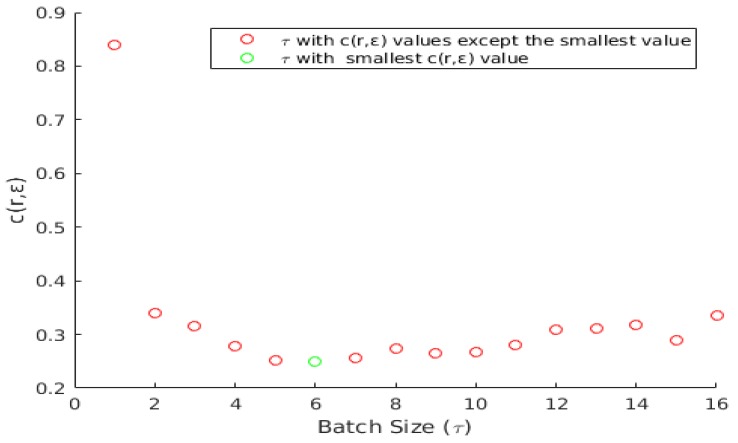
An example plot of cost function $c(r_{\tau },\varepsilon_{\tau })$ and batch size τ (τ = [1 16]).

Figure 3 indicates that frequent updates (small batch size) on the basis of the proposed method has a lower tracking error than for large batch size. The reason for this behavior is that it updates the most recent appearance of the face and also the resync (if required) of the features are performed after a specific number of frames. The optimal trade-off is the batch size that minimizes both the number of resyncs (*r*) and the tracking error ε, which is defined as:

(15) $$arg min\forall_{\tau =1}^{n}\;c\left(r_{\tau },\varepsilon_{\tau }\right),s.t.\;\;c\left(r_{\tau },\varepsilon_{\tau }\right)=\left(1-\kappa \right)r_{\tau }+\kappa \varepsilon_{\tau },$$

where *c* indicates a cost function, *n* is the total number of batch sizes (*n* = 16 in the current experiments), and κ is a bias between the tracking error ε and number of resyncs *r* (κ = 0.5 in the current experiments). Figure 4 shows an example graph of the batch size and the cost function. The objective is to minimize the cost function to achieve an optimal batch size (τ) and in this example the error function attains minimum value (green circle) when τ is 6.

### 3.2. Discussion on Error Prediction and Resyncing

**Figure 5:**
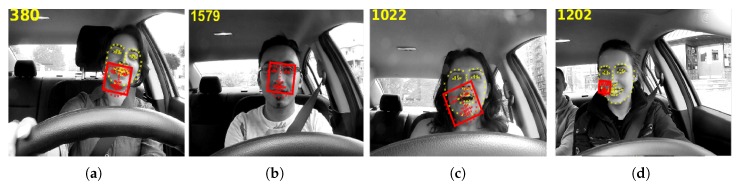
Failed results in challenging conditions using the ILFT method: (**a**) tracking failure due to face tilting; (**b**) bad lighting leading to error; (**c**) face expression leading to error (**d**) tracking failure due to similarity with the background.

Figure 5 shows some examples of the tracking errors of the proposed ILFT method without the error prediction and the resyncing procedure, illustrating some video frames with the tracked target face enclosed in a bounding box and the tracked facial landmarks plotted in red, whereas the yellow facial landmarks show the ground-truth landmarks. Figure 5a shows the effect of a tilted face on the tracking process, and Figure 5b shows that bad lighting also affects the tracking process, which tends to decrease its performance when the lighting conditions are changed during tracking. When the face deformation is not detected correctly, it is difficult to do facial expression analysis as shown in Figure 5c. The illumination changes may cause the tracked target face to be confused with the background, resulting in the tracking process permanent failure as can be seen in Figure 5d. Often, the tracking process fails in complex scenarios, since the eigenbases are then built using slightly incorrect tracked target face samples.

**Figure 6:**
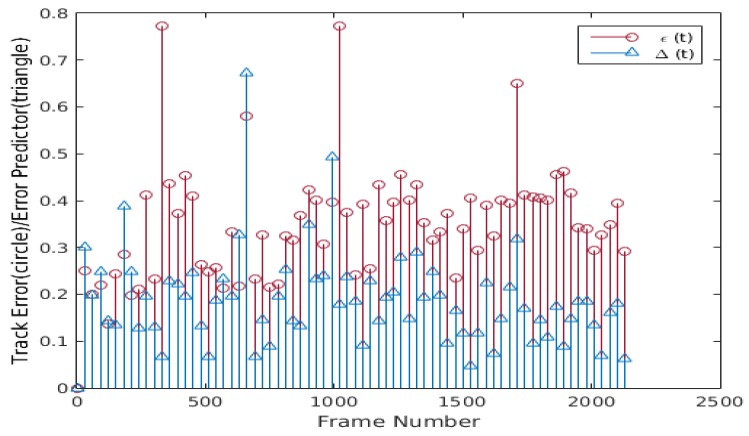
Plot of Δ(t) and ε(t) (ρ = 0.21658).

**Figure 7:**
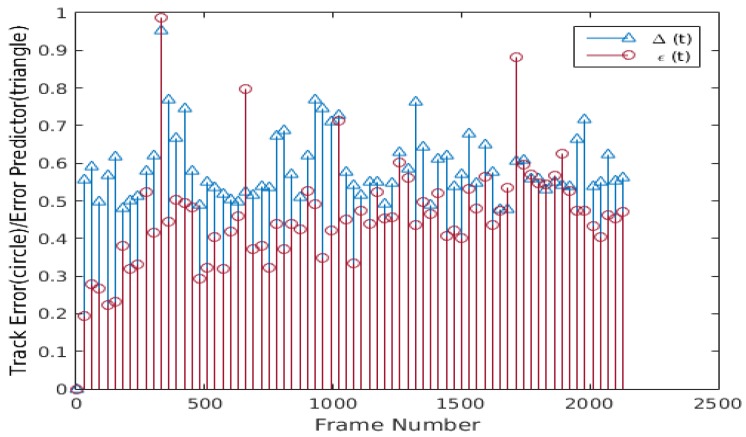
Plot of $\bar{\Delta }\left(t\right)$ and $\bar{\varepsilon }\left(t\right)$, the median of Δ(t) and ε(t) (ρ = 0.68328).

**Figure 8:**
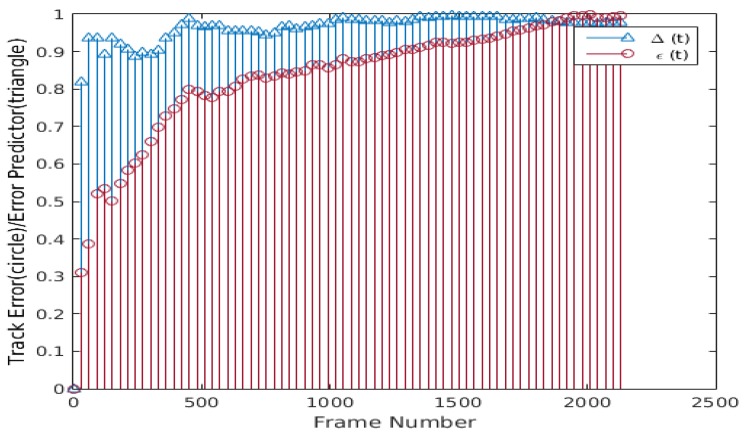
Plot of $\hat{\Delta }\left(t\right)$ and $\hat{\varepsilon }\left(t\right)$, the median of $\bar{\Delta }\left(t\right)$ and $\bar{\varepsilon }\left(t\right)$ (ρ = 0.91037).

**Figure 9:**
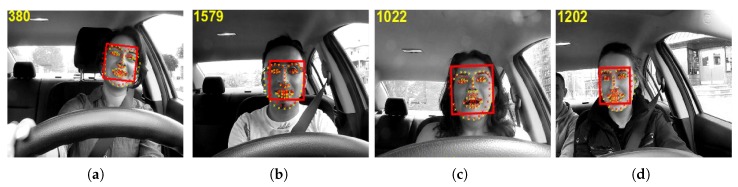
Illustration of the results obtained by the Adaptive Face Tracker with Resyncing Mechanism using the W-CLM (AFTRM-W) method: (**a**) tilted face; (**b**) bad lighting; (**c**) face expression change; and (**d**) similar background.

Nevertheless, this tracking failure can be avoided if the tracker has an estimate of the tracking error. The proposed method addresses this problem using an error predictor and a resyncing scheme. Figure 6 shows the plots of the proposed error predictor Δ(t) computed using Equation (12) and the actual tracking error ε(t) of the tracked facial landmarks. The plots in Figure 6 suggest some correlation ρ between Δ(t) and actual tracking error ε(t), but the data is noisy and the correlation is low. Due to the noisy nature of Δ(t) and the actual tracking error ε(t), a one dimensional median filter of fifth order is applied on a sliding window of τ frames to smooth consistently Δ(t) (i.e., $\bar{\Delta }\left(t\right)$={Δ(t)−τ, Δ(t)−τ+1, …, Δ(t)}), increasing the correlation between $\bar{\varepsilon }\left(t\right)$ and $\bar{\Delta }\left(t\right)$, as shown in Figure 7. It can be seen that the filtered $\bar{\Delta }\left(t\right)$ and $\bar{\varepsilon }\left(t\right)$ have higher correlation because the data is smoothed and has fewer spikes. To further improve the tracking error prediction, a median filter of fifth order is applied over a sliding window of τ previous values of $\bar{\Delta }\left(t\right)$ (i.e., $\hat{\Delta }\left(t\right)$={$\bar{\Delta }\left(t\right)-\tau$, $\bar{\Delta }\left(t\right)-\tau +1$, …, $\bar{\Delta }\left(t\right)$}), and the correlation between $\hat{\Delta }\left(t\right)$ and $\hat{\varepsilon }\left(t\right)$ is improved, as can be seen in Figure 8. Using the proposed error predictor, the tracking quality can be evaluated and the re-estimation of the tracking landmarks locations uses W-CLM when Ψ(t) = 1 (see Equation (13)). Some results obtained using this error prediction and resyncing based face tracking scheme are shown in Figure 9. The proposed tracking process tends to adapt to the changes in the tracked target face and work correctly in long video sequences, even if there is a tilt in the face (see Figure 9a), bad lighting (see Figure 9b), changes in face expression (see Figure 9c), or if the tracked face is similar to the background and under varied face expressions (see Figure 9d).

### 3.3. Quantitative Evaluation of the Proposed Face Tracking Method

Next is presented a quantitative comparison of the proposed AFTRM and AFTRM-W with the following methods: Incremental Learning Tracking Based on Independent Component Analysis (ILICA) [5], Incremental Learning for Robust Visual Tracking (IVT) [7], Incremental Cascaded Continuous Regression (iCCR) [15], Approximate Structured Output Learning for CLM [30], MCVFT [11], DCFNet [13], and MMDL-FT and MMDL-FTU [31].

Table 3 shows the RMSE in tracking of the facial landmarks of the proposed AFTRM, AFTRM-W, and of the comparative methods. Each column indicates the average RMSE tracking error $\varepsilon_{m}$ for the whole video sequence using the method specified in the first column. The last column illustrates the average tracking error obtained for all the tested videos. For the comparative methods, the parameters (if required) are set to the default values as proposed by their respective authors. Furthermore, the initialization for Terissi et al. [5], Ross et al. [7], Wang et al. [13], Li et al. [11], MMDL-FT, MMDL-FTU [31], AFTRM and AFTRM-W is done by using the W-CLM search method. Furthermore, Table 4 compares the CLE of the proposed AFTRM-W with the state-of-the-art methods based on all the videos of YawDD dataset with the camera installed on dash. Table 3 and Table 4 show that the proposed AFTRM and AFTRM-W tend to outperform the other methods, whereas AFTRM-W has an improved performance in comparison with AFTRM. This is due to the weighting scheme, as consistent landmarks receive higher weights, improving the quality of the resyncing mechanism. The methods proposed by Zheng et al. [30], Sanchez et al. [15], Wang et al. [13] and our previous MMDL-FTU method [31] perform similarly to the proposed AFTRM method, whereas AFTRM-W has performed better than all the other tested methods and has a smaller tracking error. In our view, the higher tracking error presented by the comparative methods occur because once the tracking error is introduced, it keeps on increasing and eventually the tracking process fails. On the other hand, we solve this problem by estimating the tracking error during tracking and resyncing the facial landmarks if the tracking error tends to increase. For this reason, the proposed method can adapt to the challenging conditions and avoid to miss the tracked target indefinitely. Consequently, the proposed method can be used for consistent face tracking and facial features tracking in long video sequences, which can be used to detect different facial expressions, such as yawning, talking, fatigue, and so on.

To compliment the experiments, the proposed method is tested on the Talking Face video [29]. Table 5 compares the proposed method with the comparative methods using CLE and RMSE measures. The experimental results show similar trend. The proposed and the comparative methods perform well on the talking face video [29]. Ross et al. [7] has performed much better on the talking face video because of its effectiveness in static background conditions. Furthermore, AFTRM-W performs better than all the comparative methods. This proves the efficiency of the proposed method and its effectiveness in the face tracking.

Face and facial landmarks can be used as a cue to many facial analysis applications, such as yawning detection, talking, facial expression detection and so on. In this paper, we evaluate the effectiveness of the proposed face and facial landmarks tracking in the context of yawning detection, which is explained next.

### 3.4. Evaluation of the Proposed Face Tracking Method in Yawning Detection

**Figure 10:**
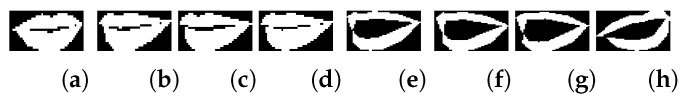
Illustration of a closed mouth and yawning sequence.

The accurate detection of facial landmarks is requirement fro many facial analysis applications such as human emotion analysis, fatigue detection and so on. To prove the effectiveness of the facial landmark features detected using the proposed tracker in a facial analysis application, i.e., a yawning detection scheme. An accurate yawning detection is requirement for many facial analysis applications. One of the most common usage of yawning is in driver fatigue detection, which is one important factor among others to detect fatigue in drivers [3]. Yawning detection is used as a case study to evaluate proposed tracking method in a practical facial tracking problem, where the local face appearance is changing. The proposed method takes an inspiration from the Omidyeganeh et al. [3] yawning detection approach, which is based on the backprojection theory and detects yawning based on the pixels counts in the binary mouth blocks of the current and the reference frames. To convert into a binary image, the pixel values greater than a threshold $\Gamma_{0}$ receive a value of 1 (named ‘white pixels’), and 0 (named ‘black pixels’) otherwise. The proposed method improves on the method proposed by Omidyeganeh et al. [3] in two ways. Firstly, the proposed method uses only the pixels which are in the lips to measure the mouth openness in a binary image (see in Figure 10 that only the pixels inside the white region are used), as compared to [3] which uses a rectangular mouth block and includes some pixels outside the lips to detect yawning. Secondly, yawning is detected in each video frame if the following three conditions are satisfied: (1) the ratio of the number of black pixels in the current frame (NBC) and the reference frame (NBR) is greater than $\Gamma_{1}$ (i.e., $\frac{NBC}{NBR}>\Gamma_{1}$); (2) the ratio of the number of black and white pixels (NWC) in the current frame is greater than $\Gamma_{2}$ (i.e., $\frac{NBC}{NWC}>\Gamma_{2}$); and (3) the ratio of a vertical distance between the midpoints (VD) and the distance between the corner points (HD) of the mouth is greater than $\Gamma_{3}$ (i.e., $\frac{VD}{HD}>\Gamma_{3}$). The first frame is used as a reference in the proposed scheme and is assumed to contain a closed mouth. Using the pixels of the reference frame tends to minimize scale issues when using conditions 2 and 3, that use only the pixels within the mouth region of the current frame. The proposed yawning detection scheme is evaluated in terms of; (1) True Positive Rate (TPR), which is the rate of True Positives (TP) detected as yawning, i.e., $TPR=\frac{TP}{TP+FN}$; True Negative Rate (TNR), which is the rate of True Negatives (TN) correctly detected as non-yawning, i.e., $TNR=\frac{FP}{FP+TN}$; False Positive Rate (FPR) is the rate of yawning falsely detected as non-yawning, False Negative Rate (FNR) is the rate of non-yawning falsely detected as yawning, and the Correct Detection Rate (CDR) is defined as $CDR=\frac{TPR+TNR}{TPR+TNR+FPR+FNR}$. Table 6 shows a comparison of the proposed method using data provided by AFTRM and AFTRM-W, with state of the art methods in yawning detection, including Chiang et al. [32], Bouvier et al. [33] and Omidyeganeh et al. [3]. The proposed method tends to outperform the comparative methods on the YawDD dataset [28]. Furthermore, the proposed method has a higher TPR, which indicates the effectiveness of the proposed method. The threshold values for $\Gamma_{1},\Gamma_{2}$ and $\Gamma_{3}$ are set to 1, 0.5 and 2.5, respectively.

## 4. Conclusions

A new adaptive face tracking scheme has been proposed, which tends to reduce the face tracking errors by using a tracking divergence estimate and a resyncing mechanism. This resyncing mechanism locates adaptively the tracked facial features (e.g., facial landmarks), which tends to reduce the tracking errors and to avoid missing the tracked face indefinitely. The proposed Weighted Constrained Local Model (W-CLM) method improves the CLM feature search mechanism by assigning higher weights to more robust facial landmarks, and is used in resyncing.

The performance of the proposed face tracking method was evaluated in the drivers video sequences of the YawDD and on Talking video datasets. Both the datasets contain significant changes in illumination and head positioning. Our experiments suggest that the proposed face tracking scheme potentially can perform better than comparable state-of-the-art methods, and can be applied in yawning detection while obtaining higher Correct Detection Rates (CDRs) and True Positive Rates (TPRs) than comparable methods available in the literature. In the future, we intend to extend our work to develop a tracker for a more general class of non-rigid objects. 

## Figures and Tables

**Table 1:** Parameters used and their ranges.

Param	min	max	Optimal
Batch Size τ	1	16	5
Face size *u*	16	64	32
Patch size *v*	4	32	8
Forgetting factor *f*	0.5	1.0	0.95

**Table 2:** Average frames per second (fps) and number of times resync is activated for different batch sizes τ in AFTRM and AFTRM-W total of 2000 frames.

Method	Batch Size (τ)									
	1	2	3	4	5	6	7	8	9	10
AFTRM	0.09	0.21	0.25	0.31	0.29	0.38	0.53	0.62	0.79	0.98
AFTRM-W	0.07	0.23	0.20	0.23	0.33	0.45	0.56	0.54	0.83	1.10
N° of resync	1002	474	279	231	194	171	161	146	*117*	**108**

**Table 3:** Comparison of facial feature tracking methods in terms of RMSE on YawDD dataset (the best results are in bold).

Video	1	2	3	4	5	6	Average
Terissi et al. [5]	38.43	26.93	50.38	66.44	66.12	16.75	34.24
Ross et al. [7]	21.43	10.56	183.7	30.12	6.23	12.17	44.04
Zheng et al. [30]	33.93	11.46	12.41	17.05	12.26	14.02	16.86
Sanchez et al. [15]	16.42	11.48	10.33	22.07	14.49	9.84	14.10
Li et al. [11]	23.14	11.20	15.58	21.46	14.97	9.61	14.74
Wang et al. [13]	20.32	11.35	**7.51**	10.84	15.33	18.07	13.57
MMDL-FT [31]	10.12	7.19	7.63	22.02	8.06	30.75	14.29
MMDL-FTU [31]	*9.73*	*6.50*	*7.76*	16.62	7.76	19.37	11.29
AFTRM	15.01	9.22	13.78	15.31	5.91	7.53	11.12
AFTRM-W	**6.54**	**3.56**	10.65	**5.27**	**4.85**	**3.62**	**5.65**

**Table 4:** Center Location Error (CLE) comparison of AFTRM and AFTRM-W with comparative methods on YawDD dataset (the best results are in bold).

Video	Male Videos	Female Videos	Average
Terissi et al. [5]	25.92	18.37	22.15
Ross et al. [7]	14.74	11.33	13.03
Zheng et al. [30]	13.02	10.14	11.58
Sanchez et al. [15]	14.11	10.17	12.14
Li et al. [11]	17.23	14.93	16.08
Wang et al. [13]	15.52	13.58	14.55
MMDL-FT [31]	10.61	8.70	9.65
MMDL-FTU [31]	10.36	8.68	9.52
AFTRM	*8.81*	*7.54*	*8.18*
AFTRM-W	**5.31**	**4.24**	**4.78**

**Table 5:** Center Location Error (CLE) and RMSE comparison of AFTRM and AFTRM-W with comparative methods on Talking Face Video datasets (the best results are in bold).

Dataset Name	Talking Face Video	
**Method**	**CLE**	**RMSE**
Terissi et al. [5]	27.79	26.43
Ross et al. [7]	12.05	10.09
Zheng et al. [30]	11.31	11.15
Sanchez et al. [15]	10.42	10.51
Li et al. [11]	9.27	11.39
Wang et al. [13]	11.63	13.46
MMDL-FT [31]	16.45	15.91
MMDL-FTU [31]	13.93	13.48
AFTRM	*8.92*	*8.98*
AFTRM-W	**6.81**	**6.62**

**Table 6:** Yawning detection results on the YawDD dataset [28].

Method	TPR	TNR	FPR	FNR	CDR
Chiang et al. [32]	0.3990	0.4562	0.6010	0.5438	0.4276
Bouvier et al. [33]	0.6764	0.5437	0.3236	0.4563	0.6101
Omidyeganeh et al. [3]	0.6578	0.7733	0.3419	0.2266	0.7155
AFTRM (Proposed)	0.8120	0.7222	0.1879	0.2777	0.76703
AFTRM-W (Proposed)	0.9307	0.7551	0.0693	0.2449	0.8429
